# Uncovering clinical and radiological asymmetry in progressive supranuclear palsy—Richardson’s syndrome

**DOI:** 10.1007/s10072-022-05919-x

**Published:** 2022-02-01

**Authors:** Marina Picillo, Maria Francesca Tepedino, Filomena Abate, Sara Ponticorvo, Roberto Erro, Sofia Cuoco, Nevra Oksuz, Gianfranco Di Salle, Francesco Di Salle, Fabrizio Esposito, Maria Teresa Pellecchia, Renzo Manara, Paolo Barone

**Affiliations:** 1 Center for Neurodegenerative Diseases (CEMAND), Department of Medicine, Surgery and Dentistry, Neuroscience Section, University of Salerno, Fisciano, Italy; 2 Department of Medicine, Surgery and Dentistry, Scuola Medica Salernitana, University of Salerno, Baronissi, (SA) Italy; 3 Department of Neurology, Mersin University School of Medicine, Mersin, Turkey; 4 Scuola Superiore Sant’Anna, Pisa, Italy; 5 Department of Diagnostic Imaging, University Hospital A.O.U. OO.RR. San Giovanni Di Dio E Ruggi D’Aragona, Scuola Medica Salernitana, Salerno, Italy; 6 Department of Neurosciences, University of Padua, Padua, Italy

**Keywords:** Progressive supranuclear palsy, Symmetry, Dystonia, Cortico-basal syndrome, Richardson’s syndrome

## Abstract

**Background:**

Richardson’s syndrome (RS) is considered the most symmetric phenotype of progressive supranuclear palsy (PSP) as opposed to PSP with predominant corticobasal syndrome (PSP-CBS) or parkinsonism (PSP-P).

**Objectives:**

Evaluate asymmetrical motor and higher cortical features in probable PSP-RS and compare the degree of asymmetry of cortical lobes and hemispheres between PSP-RS, PSP-CBS, PSP-P, and age-matched healthy controls (HC).

**Methods:**

Asymmetry of motor and higher cortical features evaluated with an extensive videotaped neurologic examination was investigated in 28 PSP-RS, 8 PSP-CBS, and 14 PSP-P. Brain MRI to compute the laterality index (LI) was performed in 36 patients as well as in 56 HC.

**Results:**

In PSP-RS, parkinsonism was the most common asymmetric motor feature (53.6%), followed by dystonia and myoclonus (21.4% and 17.9%, respectively). Among higher cortical features, limb apraxia was found asymmetric in about one-third of patients. PSP-RS disclosed higher LI for hemispheres compared to HC, indicating a greater degree of asymmetry (*p* = 0.003). The degree of asymmetry of clinical features was not different between PSP-RS and those qualifying for PSP-CBS or PSP-P. As for imaging, LI was not different between PSP-RS, PSP-CBS, and PSP-P in any cortical region.

**Conclusions:**

Motor and higher cortical features are asymmetric in up to 50% of PSP-RS who also present a greater degree of asymmetry in hemispheres compared to age-matched HC. Lateralization of clinical features should be annotated in PSP.

## Introduction

RS is the most common phenotype of PSP and is characterized by the early onset of postural instability and falls and vertical supranuclear gaze palsy [1]. Traditionally, and as opposed to other phenotypes of disease, PSP-RS has been considered a symmetric disease [2–4]. Indeed, prominent asymmetric clinical features prompt clinicians to consider either PSP-CBS or PSP-P [1]. On a clinical ground and given the lack of biomarkers, the correct identification of the latter phenotypes often represents a clinical challenge and, despite the rigorous application of clinical diagnostic criteria, the final diagnosis is often based on clinical judgment [1, 5].

Although current clinical diagnostic criteria for probable PSP show high sensitivity and specificity, definite PSP remains a neuropathological diagnosis as in vivo biomarkers are lacking [1]. Despite the adequate diagnostic accuracy of the operational algorithm suggested, the different phenotypes of the disease show massive overlap and, thus, clinical classification often represents a difficult work [1, 5, 6]. Furthermore, patients classified as PSP-RS can show asymmetric clinical features or have a neuropathologic diagnosis of corticobasal degeneration [7, 8]. Likewise, patients showing asymmetrical clinical features can present neuropathology typical for PSP [9].

As the PSP rating scale, the most frequently used tool to rate disease severity in PSP, does not value lateralization, detailed information on the asymmetry of clinical features in PSP-RS is lacking [10]. Similarly, no evidence is available on the degree of asymmetry of cortical regions in PSP-RS compared to PSP-CBS and PSP-P or healthy controls (HC).

Objectives of the present retrospective chart review were to (1) systematically evaluate asymmetrical motor and higher cortical features in a cohort of probable PSP-RS diagnosed according to current clinical criteria in comparison with the other variant syndromes of PSP (i.e., PSP-CBS and PSP-P); (2) compare the degree of asymmetry of cortical lobes and hemispheres between PSP-RS and age-matched PSP-CBS and PSP-P and HC; (3) explore correlations between clinical and radiological asymmetry.

## Methods

### Patients and clinical evaluation

Twenty-eight patients with probable PSP-RS, 8 with possible PSP-CBS, and 14 with probable PSP-P according to the Movement Disorder Society (MDS) criteria were included in the present analysis [1]. Detailed information on enrollment and application of the PSP diagnostic criteria is available elsewhere [6, 11–13]. All assessments were videotaped. Briefly, 83 PSP outpatients were enrolled from the Movement Disorders Centers of the University of Salerno between November 2015 and December 2019. Thirty-three patients were excluded because they either had incomplete data (*N* = 7) or qualified for other phenotypes other than probable or possible PSP-RS and PSP-P or possible PSP-CBS by applying MDS guidelines (*N* = 26) [1, 5]. Excluded patients had a median (interquartile age) of 68 (8) years. Eighteen (54.5%) were men, and the median disease duration was 2 (3) years.

Motor features, including bradykinesia, rigidity, rest tremor, and dystonia were evaluated with specific items from the Neuroprotection and Natural History in Parkinson Plus Syndromes (NNIPPS) scale [14]. Any features presenting a different scoring between the right and left side were deemed as asymmetric: bradykinesia, rigidity, tremor, and dystonia (corresponding NNIPPS items are detailed in Table 1). As NNIPPS provides no information on lateralization for myoclonus, such a feature was excluded from the present analysis.

**Table 1:** Frequency of asymmetric scoring for motor and higher cortical features in PSP-RS with side predominance

		Patients with asymmetric scoring (%)	*Predominance (R/L) (%)
NNIPPS item	Motor features		
*Limb bradykinesia*	*Bradykinesia*		
4.1/4.2	Finger tapping	16 (57.1)	4/12 (25/75)
4.3/4.4	Hand movements	15 (53.6)	4/11 (26.6/73.4)
4.5/4.6	Rapid alternating hand movements	16 (57.1)	5/11 (31.2/68.7)
4.7/4.8	Leg agility	14 (50)	4/10 (28.5/71.4)
*Rigidity*	*Rigidity*		
3.2/3.3	Upper limbs rigidity	16 (57.1)	7/9 (43.7/56.2)
3.4/3.5	Lower limbs rigidity	13 (46.4)	5/8 (38.4/61.5)
*Tremor*	*Tremor*		
3.3/3.4	Upper limbs rest tremor	4 (14.3)	2/2 (50/50)
*Axial and limb dystonia*	*Dystonia*		
5.1/5.2	Upper limbs dystonia	5 (17.9)	2/3 (40/60)
5.3/5.4	Lower limbs dystonia	3 (10.7)	0/3 (0/100)
	Higher cortical features		
NA	Limb apraxia	10 (35.7)	4/6 (40/60)

Abbreviations: *only in patients with asymmetric scoring; *NNIPPS*, Neuroprotection and Natural History in Parkinson Plus Syndromes

As for higher cortical features, limb apraxia was explored with the ideomotor apraxia test for each side [6, 15]. The test consists of 24 tasks, 12 concern symbolic gestures, and 12 nonsymbolic gestures; each task is performed for a maximum of three times, and the maximum score is 72. In accordance with the Italian correction criteria, raw scores less than 53 were considered pathological; scores between 53 and 62 were considered borderline, i.e., representative of a probable but uncertain diagnosis, and all scores above 62 were considered within the normal range [6, 15]. Any different ranking between right and left was deemed as asymmetric. Alien limb phenomenon was not included in the evaluation, as no objective score was administered. Of note, none of the patients presented bilateral alien limb phenomena.

Furthermore, for each clinical feature, the degree of asymmetry was calculated with the percentage ratio between the absolute value of the difference between left and right score and the sum of them, as follows: LI = ((left − right)/(left + right)) × 100, where lower LI values indicate a decrease of the degree of asymmetry for the specific feature, i.e., LI = 0 when left = right. Thus, in this work, the LI represents an asymmetry measure not directed toward one particular side of the body. Right or left predominance was attributed by reviewing the scores within each item (i.e., a greater scoring on the right was interpreted as right predominance).

### MRI imaging protocol

Brain MRI was performed in all PSP-CBS, a subset of 17 PSP-RS and 11 PSP-P patients and in 56 age-matched HC (median age (interquartile range) 69 (10)] on a 3 T system (Magnetom Skyra, Siemens, Erlangen, Germany). A volumetric 3D T1-weighted magnetization prepared rapid gradient echo (MPRAGE) sequence was acquired with the following parameters: repetition time = 2400 ms, echo time = 2.25 ms, resolution = 1 × 1 × 1 mm^3^, matrix size = 256 × 256, 192 sagittal slices, anterior–posterior phase-encoding direction, generalized autocalibrating partially parallel acquisition (GRAPPA) factor of 2 in phase-encoding direction. MPRAGE images were processed using FreeSurfer version 6.0 (https://surfer.nmr.mgh.harvard.edu/) using the standard structural image preprocessing and surface reconstruction pipeline via the “recon-all" command (for a detailed description of this procedure, please see https://surfer.nmr.mgh.harvard.edu/fswiki/ReconAllTableStableV5.3) [16]. Preprocessed data were visually inspected to assure the quality of each reconstruction. Cortical volume was extracted for each region of the Desikan–Killiany cortical atlas, and then regions were merged (and volumes were summed) to obtain one regional value for each cerebral lobe (frontal, parietal, occipital, temporal, and cingulate) as well as for each hemisphere [16, 17]. The total intracranial volume was also considered and compared between groups.

The degree of asymmetry for each cerebral lobe as well as hemispheres was calculated with the percentage ratio between the absolute value of the difference between left and right raw volume (not normalized by intracranial volume) and the sum of them, as follows: LI = ((left − right)/(left + right)) × 100, where lower LI values indicate a decrease of the degree of asymmetry in the specific structure, i.e., LI = 0 when left = right. Thus, in this work, the LI represents an asymmetry measure not directed toward one particular hemisphere.

### Standard protocol approvals, registrations, and patient consent

The project was approved by the local ethics committee, and each subject was included after signing the informed consent form.

### Statistical analysis

After checking normality distribution with the Kolmogorov–Smirnov test, group comparisons were run with *χ*^2^, Mann–Whitney, or Kruskal–Wallis test as appropriate. Correlation analysis was performed with the Spearman’s test. All *p*-values were 2-tailed, and the significance threshold was set at ≤ 0.05. Post hoc analysis was run with the Bonferroni test. The statistical analysis was conducted using SPSS 23.0 (IBM, Chicago, IL, USA).

## Results

### Asymmetric motor and higher cortical features in PSP-RS

Enrolled patients had a median (interquartile range) age of 71 (10) years. Thirteen (46.4%) were men, and the median disease duration was 3 (3) years. More than 95% complained of falls at the onset of disease. The frequency of asymmetry with side predominance for each asymmetric motor and higher cortical feature is detailed in Table 1.

As for motor symptoms, bradykinesia and rigidity were asymmetric in more than 50% of the cohort, while the rest tremor only in 14.3%. Parkinsonism was the most common asymmetric motor feature (53.6%), followed by dystonia and myoclonus (21.4% and 17.9% each).

As for higher cortical features, limb apraxia was found asymmetric in about one-third of patients (35.7%), while alien limb was not present in any patient.

### Comparison between PSP-RS, PSP-CBS, and PSP-P

**Fig. 1:**
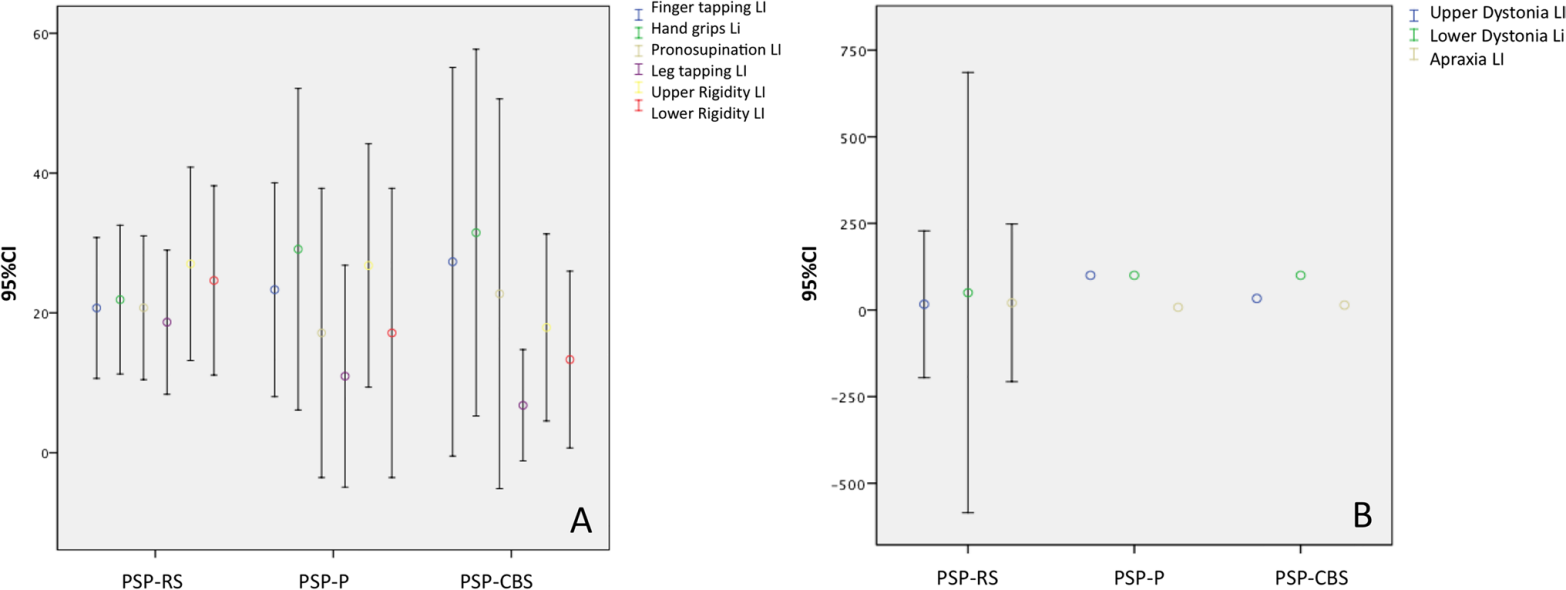
**A** Comparison between PSP-RS, PSP-P, and PSP-CBS of the laterality index (LI, 95% confidence intervals) for bradykinesia and rigidity; **B** comparison between PSP-RS, PSP-P, and PSP-CBS of the laterality index (95% confidence intervals) for upper and lower limbs dystonia and limb apraxia. Abbreviations: CI, confidence interval; PSP-CBS, progressive supranuclear palsy with cortcobasal syndrome; PSP-P, progressive supranuclear palsy with predominant parkinsonism; PSP-RS, progressive supranuclear palsy with Richardson’s syndrome

Demographic and clinical features in PSP-RS compared to PSP-CBS and PSP-P are detailed in Table 2. LI for each clinical feature was not different between groups (*p* > 0.05) (Fig. 1).

**Table 2:** Demographic and clinical features in PSP-RS versus PSP-CBS and PSP-P

	PSP-RS (*N* = 28)	PSP-CBS (*N* = 8)	PSP-P (*N* = 14)	*p*
Sex, men, *N* (%)	13 (46.4)	4 (50)	12 (85.7)	0.045^*^
Age, years	71 (10)	72 (14)	68.5 (9)	0.414
Disease duration, years	3 (3)	4 (4)	2 (4)	0.354
Falls at onset, *N* (%)	27 (96.4)	6 (75)	7 (50)	0.001^§^

Data are shown in median (interquartile range) unless otherwise specified

Significant results are in bold

Abbreviations: *PSP-CBS*, progressive supranuclear palsy with predominant corticobasal syndrome; *PSP-RS*, progressive supranuclear palsy with Richardson’s syndrome

^*^Men were more represented in PSP-P compared to PSP-RS (*p* = 0.014)

^§^Falls at onset were more frequent in PSP-RS compared to PSP-P (*p* < 0.001)

### Degree of asymmetry of cortical lobes and hemispheres: comparison between PSP-RS, PSP-CBS, PSP-P, and HC

The volume of cerebral lobes was not normalized by intracranial volume, as this was not different between groups (*p* = 0.706).

**Fig. 2:**
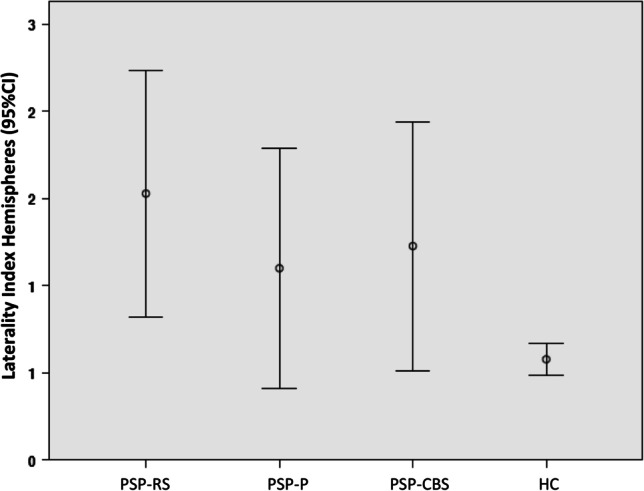
Hemisphere Laterality Index (95% confidence intervals) in PSP-RS, PSP-P, PSP-CBS, and HC. PSP-RS shows greater laterality index compared to HC (*p* = 0.003). Abbreviations: CI, confidence interval; HC, healthy controls; PSP-CBS, progressive supranuclear palsy with cortcobasal syndrome; PSP-P, progressive supranuclear palsy with predominant parkinsonism; PSP-RS, progressive supranuclear palsy with Richardson’s syndrome

Kruskal–Wallis showed LI for hemispheres was different between groups (*p* = 0.006) with PSP-RS presenting a greater degree of asymmetry compared to HC (*p* = 0.003) (Fig. 2). No other difference in LI was detected for other lobes (all *p* > 0.05).

### Correlation between clinical and radiological asymmetry

Spearman’s test did not disclose any significant correlation between clinical and radiological LI (*p* > 0.05).

## Discussion

This is the first systematic assessment of asymmetry in motor and higher cortical features in PSP-RS. Contradicting previous evidence [2–4], we showed that parkinsonism, dystonia, and myoclonus are asymmetric in a relevant proportion of PSP-RS (53.6%, 21.4%, and 17.9% each). In a lower proportion of patients, asymmetry was revealed also for higher cortical features such as limb apraxia (35.7%).

A large body of literature refers to PSP-RS as a symmetric disease [2–4]. As a result, the most widely used instrument to assess disease severity and progression in the context of clinical trials, the PSP rating scale, lacks a scoring system reporting asymmetry of clinical features [10]. Similarly, the PSP Clinical Deficits Scale recently released by the MDS does not provide any information on lateralization in the akinesia-rigidity and finger dexterity functional domains [18]. Notwithstanding, in line with previous reports also including pathologically proven cases, our data show that asymmetry can be present in PSP-RS [7–9].

Asymmetric clinical features in our cohort of PSP-RS were detected with the application of a rigorous protocol, including videotaped NNIPPS and neurological examination as well as the ideomotor apraxia test. Of note, the consistency of our results is supported by several findings. First, the different tasks evaluating bradykinesia presented a similar rate of asymmetry (Table 1, Fig. 1). Second, bradykinesia and rigidity, the two most relevant parkinsonian features, also showed similar rates of asymmetry. Asymmetry rates were higher in the upper compared to lower limbs. An assessment bias with a greater number of items evaluating the upper limbs may account for such discrepancy. Alternatively, we can not exclude a greater rate of asymmetry in the upper, compared to lower limbs.

The relevance of clinical asymmetry was further supported by the evidence of a significant degree of morphological cortical hemispheric asymmetry in PSP-RS compared to age-matched HC but not compared to the other variant syndromes of PSP (Fig. 2).

Comparing clinical features of PSP-RS with patients qualifying for the other variant syndromes of PSP disclosed similar rates of asymmetry (Fig. 2). In agreement with clinical findings, the laterality index did not differ between PSP-RS and those qualifying for PSP-CBS or PSP-P in any cortical region. Taken together, our findings suggest asymmetry should not be mentioned among major features differentiating PSP-RS from PSP-CBS and PSP-P.

Our data have practical implications. The distribution of clinical features should not be considered in support of the phenotypic characterization of PSP patients (i.e., symmetric in PSP-RS versus asymmetric in PSP-CBS and PSP-P). Furthermore, clinical evaluation of PSP patients should also include ratings of motor and cortical features for each side of the body. Given the lack of reliable radiological markers [12], our study further confirms the difficulty in differentiating PSP clinical phenotypes as theorized by the MDS [6, 10].

Our study has limitations. First, we recognize the lack of pathological confirmation of both diagnosis and phenotypic categorization, which remains the gold standard for PSP diagnosis. However, the MDS diagnostic flow chart and phenotypic attribution have been applied rigorously by experts in movement disorders, all patients underwent detailed clinical examination and imaging ruled out vascular lesions as well as signs suggesting the presence of normal pressure hydrocephalus [12]. We also acknowledge some patients may have evolved into PSP-RS after having started out as PSP-P or PSP-CBS. However, we enrolled a population of early PSP-RS patients (median disease duration = 3 years), and more than 95% complained of falls at onset of disease, further corroborating PSP-RS was the starting phenotype. As a second limitation, we acknowledge our cohort only included a cross-sectional evaluation. However, all patients but those qualifying for PSP-CBS already reached a degree of diagnostic certainty of probability. Also, we did not evaluate the dopamine transporter SPECT imaging asymmetry index as enrolled patients did not perform such exams systematically at our center. Furthermore, our results should be interpreted with caution due to the small sample size of PSP phenotypes. Finally, we also recognize the lack of evaluation of cortical sensory loss among the higher cortical features. We performed the regional analysis using the Desikan–Killiany cortical atlas (and then the lobe parcellation), the standard parcellation included in the Freesurfer framework as it is the most used in morphological analysis and it is adapted to every subject’s anatomy. Nevertheless, we recognize we may have missed subtle asymmetries or small alterations considering the big sizes of the regions.

In conclusion, the present study demonstrates that motor and higher cortical features are asymmetric in a relevant proportion of PSP-RS (up to 50%). PSP-RS patients also present a greater degree of asymmetry in hemispheres compared to age-matched HC but not compared to the other variant syndromes of PSP. The distribution of clinical features should be considered in the clinical characterization of PSP patients.

## Data Availability

The dataset of the present study is available from the corresponding author upon request.

## Glossary

HC: Healthy controls
LI: Laterality index
MDS: Movement Disorder Society
MRI: Magnetic resonance imaging
PSP: Progressive supranuclear palsy
NNIPPS: Neuroprotection and Natural History in Parkinson Plus Syndromes
PSP-CBS: Progressive supranuclear palsy with predominant corticobasal syndrome
PSP-P: Progressive supranuclear palsy with predominant parkinsonism
RS: Richardson’s syndrome
